# Supplementary material for: MEME-LaB: motif analysis in clusters
Source: Bioinformatics. 2013 May 14;29(13):1696–7. doi: 10.1093/bioinformatics/btt248 (PMC3694638; doi:10.1093/bioinformatics/btt248)
Supplement: Supplementary Data [file supp_btt248_Supplementary_Data.zip › Supplementary_Data/Results_Files/which_gene_ids/cluster11_motif1.html]

Cluster 11 Motif 1


Cluster 11 Motif 1 is found in the following promoters

| Gene ID | Strand | Position\* |
| --- | --- | --- |

|  |  |  |
| --- | --- | --- |
| AT1G01930 | + | 298/500 |
| AT1G02150 | + | 317/500 |
| AT1G04590 | - | 123/500 |
| AT1G06240 | + | 323/500 |
| AT1G06640 | - | 279/500 |
| AT1G06950 | - | 484/500 |
| AT1G07280 | - | 401/500 |
| AT1G07720 | - | 445/500 |
| AT1G09030 | + | 486/500 |
| AT1G09760 | + | 327/500 |
| AT1G11750 | - | 316/500 |
| AT1G11840 | + | 307/500 |
| AT1G12770 | - | 194/500 |
| AT1G13250 | + | 66/500 |
| AT1G13810 | + | 201/500 |
| AT1G14620 | + | 220/500 |
| AT1G14840 | + | 130/500 |
| AT1G15340 | + | 278/500 |
| AT1G15810 | - | 190/500 |
| AT1G15890 | + | 209/500 |
| AT1G16350 | - | 354/500 |
| AT1G16680 | - | 89/500 |
| AT1G17850 | + | 67/500 |
| AT1G19835 | - | 33/500 |
| AT1G20810 | + | 112/500 |
| AT1G21880 | - | 467/500 |
| AT1G23280 | + | 350/500 |
| AT1G23750 | - | 43/500 |
| AT1G26650 | - | 304/500 |
| AT1G29810 | - | 387/500 |
| AT1G29880 | + | 197/500 |
| AT1G31410 | + | 230/500 |
| AT1G32160 | + | 272/500 |
| AT1G32220 | - | 355/500 |
| AT1G32580 | - | 87/500 |
| AT1G33040 | - | 347/500 |
| AT1G33810 | + | 230/500 |
| AT1G35340 | + | 77/500 |
| AT1G47330 | - | 4/500 |
| AT1G47580 | - | 461/500 |
| AT1G49510 | - | 242/500 |
| AT1G49980 | + | 128/500 |
| AT1G51570 | + | 324/500 |
| AT1G52670 | + | 334/500 |
| AT1G53230 | - | 457/500 |
| AT1G53380 | - | 473/500 |
| AT1G53450 | + | 360/500 |
| AT1G53460 | + | 216/500 |
| AT1G54310 | + | 36/500 |
| AT1G54520 | + | 381/500 |
| AT1G54790 | - | 8/500 |
| AT1G55890 | - | 194/500 |
| AT1G59750 | + | 165/500 |
| AT1G60770 | + | 24/500 |
| AT1G60800 | - | 236/500 |
| AT1G60990 | - | 161/500 |
| AT1G63880 | - | 31/500 |
| AT1G64355 | + | 474/500 |
| AT1G64390 | - | 94/500 |
| AT1G64810 | - | 66/500 |
| AT1G65070 | + | 125/500 |
| AT1G66840 | - | 48/500 |
| AT1G67440 | - | 244/500 |
| AT1G68870 | + | 359/500 |
| AT1G70200 | - | 75/500 |
| AT1G70310 | - | 226/500 |
| AT1G74070 | - | 106/500 |
| AT1G74210 | + | 395/500 |
| AT1G74550 | + | 352/500 |
| AT1G74730 | + | 266/500 |
| AT1G75500 | - | 167/500 |
| AT1G75800 | + | 447/500 |
| AT1G77122 | - | 249/500 |
| AT1G77750 | + | 413/500 |
| AT1G78060 | + | 487/500 |
| AT1G79030 | - | 141/500 |
| AT2G01330 | + | 338/500 |
| AT2G01755 | + | 305/500 |
| AT2G01810 | - | 467/500 |
| AT2G02120 | + | 295/500 |
| AT2G02795 | + | 249/500 |
| AT2G03390 | - | 381/500 |
| AT2G04700 | - | 485/500 |
| AT2G04842 | + | 222/500 |
| AT2G13440 | + | 377/500 |
| AT2G13690 | + | 474/500 |
| AT2G17240 | + | 294/500 |
| AT2G17972 | + | 458/500 |
| AT2G19680 | + | 300/500 |
| AT2G20050 | - | 434/500 |
| AT2G21170 | + | 468/500 |
| AT2G21280 | + | 36/500 |
| AT2G21300 | - | 57/500 |
| AT2G21385 | - | 485/500 |
| AT2G21530 | + | 101/500 |
| AT2G21960 | + | 405/500 |
| AT2G22230 | + | 314/500 |
| AT2G22540 | - | 300/500 |
| AT2G23470 | + | 221/500 |
| AT2G24020 | - | 261/500 |
| AT2G25270 | - | 484/500 |
| AT2G26330 | - | 478/500 |
| AT2G27290 | + | 485/500 |
| AT2G27590 | + | 340/500 |
| AT2G27920 | + | 336/500 |
| AT2G28260 | - | 7/500 |
| AT2G29120 | - | 89/500 |
| AT2G29510 | - | 411/500 |
| AT2G30320 | + | 230/500 |
| AT2G31170 | - | 297/500 |
| AT2G32440 | - | 112/500 |
| AT2G32580 | - | 243/500 |
| AT2G32950 | + | 29/500 |
| AT2G33255 | + | 97/500 |
| AT2G33800 | - | 248/500 |
| AT2G33855 | - | 263/500 |
| AT2G34300 | - | 291/500 |
| AT2G34860 | + | 25/500 |
| AT2G35500 | + | 178/500 |
| AT2G35650 | + | 97/500 |
| AT2G37080 | - | 459/500 |
| AT2G38040 | - | 312/500 |
| AT2G38140 | + | 334/500 |
| AT2G38950 | - | 362/500 |
| AT2G39740 | + | 65/500 |
| AT2G40490 | + | 76/500 |
| AT2G42190 | - | 87/500 |
| AT2G43360 | - | 324/500 |
| AT2G44060 | - | 42/500 |
| AT2G44160 | - | 316/500 |
| AT2G44670 | + | 264/500 |
| AT2G45700 | - | 439/500 |
| AT2G45970 | + | 148/500 |
| AT2G47590 | - | 367/500 |
| AT3G01450 | + | 468/500 |
| AT3G03220 | + | 86/500 |
| AT3G04810 | - | 329/500 |
| AT3G05100 | + | 362/500 |
| AT3G06035 | + | 134/500 |
| AT3G06080 | + | 166/500 |
| AT3G06730 | - | 303/500 |
| AT3G07460 | + | 54/500 |
| AT3G07670 | - | 383/500 |
| AT3G08010 | + | 89/500 |
| AT3G09050 | + | 207/500 |
| AT3G09210 | - | 22/500 |
| AT3G10140 | + | 325/500 |
| AT3G10330 | + | 483/500 |
| AT3G10970 | + | 172/500 |
| AT3G11560 | - | 452/500 |
| AT3G12390 | + | 124/500 |
| AT3G12685 | + | 348/500 |
| AT3G13180 | + | 379/500 |
| AT3G13510 | + | 453/500 |
| AT3G13740 | + | 13/500 |
| AT3G14340 | + | 6/500 |
| AT3G14900 | + | 383/500 |
| AT3G14930 | + | 55/500 |
| AT3G15190 | + | 335/500 |
| AT3G15380 | + | 478/500 |
| AT3G15720 | - | 178/500 |
| AT3G15790 | + | 206/500 |
| AT3G17170 | + | 163/500 |
| AT3G17470 | + | 282/500 |
| AT3G18050 | + | 50/500 |
| AT3G18680 | + | 144/500 |
| AT3G19370 | + | 117/500 |
| AT3G20230 | + | 176/500 |
| AT3G21465 | + | 289/500 |
| AT3G22104 | + | 384/500 |
| AT3G22190 | - | 18/500 |
| AT3G24040 | + | 472/500 |
| AT3G24120 | + | 305/500 |
| AT3G26080 | + | 28/500 |
| AT3G26650 | - | 43/500 |
| AT3G26720 | + | 250/500 |
| AT3G28200 | - | 488/500 |
| AT3G28540 | + | 104/500 |
| AT3G28760 | - | 15/500 |
| AT3G29320 | - | 354/500 |
| AT3G32930 | + | 196/500 |
| AT3G46530 | + | 97/500 |
| AT3G46580 | - | 434/500 |
| AT3G46610 | + | 488/500 |
| AT3G49250 | - | 45/500 |
| AT3G49720 | + | 132/500 |
| AT3G50270 | - | 419/500 |
| AT3G52380 | + | 220/500 |
| AT3G53560 | - | 476/500 |
| AT3G53850 | + | 410/500 |
| AT3G55360 | + | 340/500 |
| AT3G56910 | - | 237/500 |
| AT3G58010 | - | 145/500 |
| AT3G58100 | - | 447/500 |
| AT3G61550 | - | 106/500 |
| AT3G62410 | - | 88/500 |
| AT3G63170 | - | 140/500 |
| AT3G63410 | - | 319/500 |
| AT3G63510 | + | 170/500 |
| AT4G00520 | + | 49/500 |
| AT4G02260 | - | 477/500 |
| AT4G02630 | - | 167/500 |
| AT4G03415 | - | 488/500 |
| AT4G03470 | - | 428/500 |
| AT4G09010 | + | 451/500 |
| AT4G09510 | + | 286/500 |
| AT4G09670 | + | 383/500 |
| AT4G09730 | - | 475/500 |
| AT4G11120 | + | 226/500 |
| AT4G12560 | + | 233/500 |
| AT4G13200 | - | 356/500 |
| AT4G15110 | - | 43/500 |
| AT4G15900 | + | 282/500 |
| AT4G15930 | - | 234/500 |
| AT4G16340 | + | 470/500 |
| AT4G18905 | - | 475/500 |
| AT4G19350 | + | 140/500 |
| AT4G19985 | - | 337/500 |
| AT4G21180 | - | 476/500 |
| AT4G21190 | + | 305/500 |
| AT4G21750 | - | 263/500 |
| AT4G22756 | + | 212/500 |
| AT4G22800 | + | 20/500 |
| AT4G23940 | - | 466/500 |
| AT4G24090 | - | 488/500 |
| AT4G24500 | + | 359/500 |
| AT4G24760 | + | 339/500 |
| AT4G24930 | + | 300/500 |
| AT4G26110 | + | 421/500 |
| AT4G26640 | + | 431/500 |
| AT4G27180 | + | 365/500 |
| AT4G28050 | - | 228/500 |
| AT4G28100 | - | 189/500 |
| AT4G29590 | + | 430/500 |
| AT4G31270 | + | 401/500 |
| AT4G32410 | - | 310/500 |
| AT4G32570 | + | 475/500 |
| AT4G33740 | + | 15/500 |
| AT4G34090 | - | 478/500 |
| AT4G34290 | + | 341/500 |
| AT4G35550 | + | 104/500 |
| AT4G35920 | + | 411/500 |
| AT4G36290 | - | 340/500 |
| AT5G01090 | - | 91/500 |
| AT5G02240 | + | 120/500 |
| AT5G03300 | - | 303/500 |
| AT5G05740 | + | 436/500 |
| AT5G06050 | - | 78/500 |
| AT5G06290 | - | 142/500 |
| AT5G07020 | + | 271/500 |
| AT5G08260 | + | 197/500 |
| AT5G08380 | + | 433/500 |
| AT5G08590 | + | 70/500 |
| AT5G10240 | - | 395/500 |
| AT5G11250 | - | 232/500 |
| AT5G11720 | + | 178/500 |
| AT5G13000 | - | 482/500 |
| AT5G13300 | - | 455/500 |
| AT5G13710 | - | 98/500 |
| AT5G13980 | + | 301/500 |
| AT5G14320 | + | 49/500 |
| AT5G14660 | + | 164/500 |
| AT5G14910 | + | 441/500 |
| AT5G15630 | - | 93/500 |
| AT5G16140 | + | 217/500 |
| AT5G16240 | - | 323/500 |
| AT5G16260 | + | 176/500 |
| AT5G17870 | - | 339/500 |
| AT5G18660 | - | 338/500 |
| AT5G19500 | - | 13/500 |
| AT5G20130 | - | 120/500 |
| AT5G20320 | + | 394/500 |
| AT5G20720 | + | 427/500 |
| AT5G20935 | - | 489/500 |
| AT5G22640 | - | 432/500 |
| AT5G22740 | - | 484/500 |
| AT5G25840 | - | 239/500 |
| AT5G27400 | + | 295/500 |
| AT5G38290 | - | 40/500 |
| AT5G39570 | - | 176/500 |
| AT5G39940 | + | 433/500 |
| AT5G40150 | + | 120/500 |
| AT5G41480 | + | 455/500 |
| AT5G41760 | - | 10/500 |
| AT5G42310 | + | 209/500 |
| AT5G42480 | + | 281/500 |
| AT5G42765 | + | 453/500 |
| AT5G42770 | + | 114/500 |
| AT5G44930 | - | 81/500 |
| AT5G45030 | + | 190/500 |
| AT5G45780 | - | 344/500 |
| AT5G45930 | + | 40/500 |
| AT5G46420 | - | 461/500 |
| AT5G48030 | + | 485/500 |
| AT5G49020 | + | 128/500 |
| AT5G49960 | + | 362/500 |
| AT5G50000 | + | 375/500 |
| AT5G50150 | - | 107/500 |
| AT5G50180 | + | 392/500 |
| AT5G50280 | + | 18/500 |
| AT5G50860 | + | 470/500 |
| AT5G50920 | - | 140/500 |
| AT5G51545 | + | 280/500 |
| AT5G53070 | - | 11/500 |
| AT5G53090 | - | 488/500 |
| AT5G55480 | - | 452/500 |
| AT5G57270 | - | 38/500 |
| AT5G57330 | - | 179/500 |
| AT5G57500 | - | 402/500 |
| AT5G57700 | - | 96/500 |
| AT5G58090 | + | 136/500 |
| AT5G58250 | - | 388/500 |
| AT5G58900 | - | 483/500 |
| AT5G58930 | - | 43/500 |
| AT5G62690 | - | 153/500 |
| AT5G62890 | + | 203/500 |
| AT5G63100 | - | 329/500 |
| AT5G63290 | - | 476/500 |
| AT5G63310 | + | 179/500 |
| AT5G64030 | + | 213/500 |
| AT5G64670 | + | 266/500 |
| AT5G65610 | - | 154/500 |
| AT5G66090 | + | 23/500 |
| AT5G66770 | - | 484/500 |

**\*** Overlapping sequences are merged to avoid redundancy. As a result some sequences in the final set may be longer than the specified maximum.

Distribution

+

-

Position values represent number of bases from the 5 prime end of the sequence provided to MEME and the length of that sequence. It is these figures expressed as proportions that are used for statistical analysis.

Probability value of comparison of these values to a linear distribution, p = 0.028

The motif is found 175 times on the + stand and 149 times on the - strand.

Probability value that it is evenly distributed between the two strands, p = 0.082

Move the mouse pointer over the gene ids to highlight the corresponding marker
